# Impact of exercise capacity on the long-term incidence of atrial arrhythmias in heart failure

**DOI:** 10.1038/s41598-021-98172-9

**Published:** 2021-09-21

**Authors:** Tetsuri Sakai, Atsuhiko Yagishita, Masahiro Morise, Susumu Sakama, Takeshi Ijichi, Kengo Ayabe, Mari Amino, Yuji Ikari, Koichiro Yoshioka

**Affiliations:** Department of Cardiology, Tokai University, Shimokasuya 143, Isehara, Kanagawa #259-1193 Japan

**Keywords:** Interventional cardiology, Cardiology

## Abstract

We sought to demonstrate the impact of improved peak exercise oxygen consumption (V̇O_2_) during maximal exercise testing after cardiac rehabilitation (CR) on the incidence of arrhythmias in patients with heart failure (HF). The present study comprised of 220 patients with HF, and peak V̇O_2_ was examined at 2 and 5 months after CR. Of the 220 patients, 110 (50%) had a low peak V̇O_2_ of < 14 mL/min/kg at 2 months. The peak V̇O_2_ improved in 86 of these 110 (78%) patients at 5 months after CR. During a median follow-up of 6 years, the patients with improvement in peak V̇O_2_, compared to those without peak V̇O_2_ improvement, had a lower rate of mortality (4% vs. 29%, log-rank, *P* < 0.001) and HF hospitalization (6 vs. 17%, log-rank, *P* = 0.044) and a lower incidence of new-onset atrial arrhythmias (9 vs. 27%, log-rank, *P* = 0.013), with no difference in the incidence of ventricular arrhythmias between groups (1 vs. 4%, log-rank, *P* = 0.309). The majority of deaths in the patients without an improved peak V̇O_2_ were because of cardiovascular events (73%), particularly progressive HF (55%). Early detection and management of atrial arrhythmias may improve outcomes in patients without peak V̇O_2_ improvement after CR.

## Introduction

Reduced exercise capacity is a principal symptom of heart failure (HF). Determination of peak exercise oxygen consumption (V̇O_2_) during maximal exercise testing has been used to assess exercise capacity in patients with HF. Mancini et al. demonstrated that a low peak V̇O_2_ of < 14 mL/kg/min was associated with poor prognosis in patients with severe HF (higher-risk)^1^. Improvement in exercise capacity is one of the most important effects of exercise training in cardiac rehabilitation (CR)^2–5^, leading to a better outcome in patients with ischemic HF^6–9^. Although atrial and ventricular arrhythmias contribute to the morbidity and mortality of patients with HF^3^, the impact of improvement in exercise capacity after CR on the long-term incidence of atrial and ventricular arrhythmias remains uncertain. We hypothesized that improvement in peak V̇O_2_ after CR would reduce the risk of long-term incidence of cardiac arrhythmias and mortality in patients with HF. In the present study, we sought to determine the association between the changes in exercise capacity after CR and the incidence of new-onset atrial and ventricular arrhythmias and the rate of mortality and hospitalization due to HF exacerbation during long-term follow-up.

## Methods

### Study design and participants

In this retrospective single-center study, 220 consecutive patients with HF who underwent CR at the Tokai University Hospital (Kanagawa, Japan) from April 2012 to March 2016 were included in the study. Patients with acute HF or exacerbation of chronic HF participated in the CR programs during the acute phase once the patients’ condition was stabilized with pharmacological and/or non-pharmacological interventions^4^. Patients who were not eligible for CR included those with acute coronary syndrome before non-pharmacological interventions, refractory arrhythmias, uncontrolled HF (e.g., New York Heart Association [NYHA] Class IV), and symptomatic severe aortic stenosis before surgical interventions, as well as those unable to provide consent to CR. Patients were classified into two groups: those with a low peak V̇O_2_ (higher-risk patients) and those without a low peak V̇O_2_ (lower-risk patients) at 2 months after the start of CR. Furthermore, higher-risk patients were subdivided into two groups: higher-risk patients with an improved peak V̇O_2_ at 5 months and those without improvement in the peak V̇O_2_. Improvement in the exercise capacity was defined as the increase in the peak V̇O_2_ after the CR. The clinical characteristics, the incidence of new-onset atrial arrhythmias including atrial fibrillation (AF) and atrial tachycardia, and ventricular arrhythmias including ventricular tachycardia and ventricular fibrillation, and the rate of mortality and hospitalization due to HF exacerbation were determined among higher-risk patients. The present study was approved by the Institutional Review Board for Clinical Research, Tokai University (20R-058). The board waived the condition of obtaining informed consent for study participation. All methods included in this study were carried out in accordance with relevant guidelines and regulations.

### Cardiopulmonary exercise testing

Patients underwent symptom-limited cardiopulmonary exercise testing (CPX) using a cycle ergometer (Lode, Groningen, Netherlands) with respiratory gas exchange analysis, when they were clinically stable, at 2 and 5 months during the CR program. The CPX protocol consisted of the following: 4-min rest period while sitting on the cycle ergometer; 2-min exercise at the free-wheel; and an incremental exercise phase with an increase of 10 W/min (ramp protocol). A 12-lead electrocardiogram was continuously monitored throughout the test (Nihon Kohden Corporation, Tokyo, Japan). Breath-by-breath expiratory gas analysis was measured, and measured minute ventilation, oxygen uptake (V̇O_2_), and carbon dioxide production data were stored on a computer hard disk every 6 s for off-line analysis (Minato Medical Science, Osaka, Japan). Peak V̇O_2_ was defined as the highest V̇O_2_ during exercise testing, which was expressed as a value adjusted to body weight (mL/kg/min). A low peak V̇O_2_ was defined as < 14 mL/min/kg^1^. Predicted values of V̇O_2_ was calculated as follows: peak VO2 pred = (Height—Age)*20 if male, = (Height—Age)*14 if female^10^.

### Statistical analysis

Categorical variables are expressed as absolute and relative frequencies. For categorical data, Fisher’s exact test was applied. Quantitative variables were described by their mean (± standard deviation [SD]) or by the median and interquartile range (IQR), as appropriate for their distribution, and compared using the Mann–Whitney U test. Survival curves were estimated using the Kaplan–Meier method and compared using log-rank tests. We performed univariate Cox regression analysis of clinical outcomes in association with improvement in peak V̇O_2_ with CR in higher-risk patients. A 2-tailed *p* value of < 0.05 was considered to indicate statistical significance. All statistical analyses were performed using SPSS Statistics version 23.0 (IBM Corp, Armonk, NY, USA).

## Results

### Patient characteristics

The median age of the study group was 67 (IQR, 59–74) years, including 51 women (23%); the median plasma brain natriuretic peptide level was 215.2 (IQR, 104.1–494.7) pg/ml. The majority of patients had an NYHA class I, with optimal medical therapy including angiotensin-converting enzyme inhibitors or angiotensin II receptor blockers and beta-blockers, at the 2-month time point of CR.

Of the 220 patients included, 110 had a low peak V̇O_2_ (50%) at 2 months of CR (higher-risk patients, Table 1). Compared to lower-risk patients, the proportion of female patients was higher (30% vs. 16%, *P* = *0.025*) and the median left ventricular ejection fraction was lower (49% vs. 54%, respectively, *P* = 0.019). In 117 patients with preserved ejection fraction on echocardiography, the cause of heart failure was not different between the two groups: ischemic in 69% in higher-risk patients vs. 83% in lower-risk patients (*P* = 0.086). The mean values of peak V̇O_2_ at 2 months were 11.8 ± 1.8 mL/min/kg in higher-risk patients and 17.4 ± 2.7 mL/min/kg in lower-risk patients, respectively (*P* < 0.001). The mean values of peak V̇O_2_ at 5 months were 14.2 ± 3.7 mL/min/kg in higher-risk patients and 19.0 ± 4.5 mL/min/kg in lower-risk patients, respectively (*P* < 0.001). Peak V̇O_2_ percentage of predicted value was 43.2 ± 9.8% in higher-risk patients and 57.5 ± 10.7% in the lower-risk patients, respectively (*P* < 0.001).

**Table 1:** Clinical characteristics of the enrolled patients.

	All (n = 220)	Lower-risk patients (n = 110)	Higher-risk patients (n = 110)	*P* value
Median age (IQR), years	67 (59–74)	65 (58–71)	68 (62–76)	0.110
Female sex, N (%)	51 (23)	18 (16)	33 (30)	0.025
Median body mass index (IQR), kg/m^2^	23.5 (20.8–25.2)	23.4 (20.5–25.2)	23.5 (21.0–25.4)	0.625
Median BNP level (IQR), pg/ml	215.2 (104.1–494.7)	213.9 (103.6–364.8)	217.6 (105.6–618.7)	0.294
Median left ventricular ejection fraction (IQR), %	50 (41–60)	54 (43–61)	49 (40–56)	0.019
Median estimated GFR(IQR), ml/min/1.73 m^2^	63.0 (52.0–77.8)	65.0 (56.0–75.5)	61.0 (46.0–78.3)	0.141
Peak V̇O2 at 2 months	14.6 ± 3.6	17.4 ± 2.7	11.8 ± 1.8	< 0.001
Peak V̇O2 at 5 months	16.6 ± 4.7	19.0 ± 4.5	14.2 ± 3.7	< 0.001
Peak V̇O_2_ percentage of predicted value	50.3 ± 12.5	57.5 ± 10.7	43.2 ± 9.8	< 0.001
**NYHA class -N. (%)**				0.066
I	147 (67)	80 (73)	67 (61)	
II	57 (26)	24 (22)	33 (30)	
III	16 (7)	6 (6)	10 (9)	
**Coexisting conditions, N (%)**				
Hypertension	152 (69)	81 (74)	71 (65)	0.189
Diabetes	77 (35)	39(36)	38 (35)	1.000
Atrial fibrillation	24 (11)	7(6)	17(16)	0.050
Prior thromboembolic events	5 (2)	2 (2)	3 (3)	1.000
CHADS2 score (IQR)	2 (2–3)	2 (2–3)	2 (2–3)	0.756
CHA2DS2–VASc (IQR)	4 (3–4)	4 (3–4)	4 (3–5)	0.942
Heart failure with preserved ejection fraction, N (%)	117 (53)	63 (57)	54 (49)	0.280
**Cause of heart failure, N (%)**				0.878
Ischemic	159 (72)	79 (72)	80 (73)	
Non-ischemic				
Idiopathic	31 (14)	17 (16)	14 (13)	
Valvular	30 (14)	14 (6)	16 (7)	
**Medications, N (%)**				
ACE inhibitor or ARB	200 (91)	100 (91)	100 (91)	1.000
Beta-blocker	177 (81)	90 (82)	87 (79)	0.734
Amiodarone	11 (5)	5 (5)	6 (6)	1.000
Preexisting pacemaker or CRT, N (%)	8 (4)	5 (5)	3 (3)	0.486

IQR; interquartile range, GFR; glomerular filtration rate, NYHA; New York Heart Association, ACE; angiotensin-converting enzyme, ARB; angiotensin II receptor blocker, CRT; cardiac resynchronization therapy, BNP; brain natriuretic peptide, GFR; glomerular filtration rate.

Among the 110 higher-risk patients, the peak V̇O_2_ improved in 86 (78%) at 5 months of CR. There were no differences in clinical characteristics between higher-risk patients with improvement in peak V̇O_2_ and those without (Table 2), except for the peak V̇O_2_ at 5 months (10.8 ± 1.8 vs. 15.1 ± 3.5 mL/min/kg, *P* < 0.001).

**Table 2:** Clinical characteristics of the higher-risk patients.

	Patients with improvement in peak V̇O2 (n = 86)	Patients without improvement in peak V̇O2 (n = 24)	*P* value
Median age (IQR), years	67 (60–76)	70 (65–78)	0.192
Female sex, N (%)	28 (33)	5 (21)	0.322
Median body-mass index (IQR), kg/m^2^	23.1 (20.9–25.1)	24.4 (21.5–27.2)	0.178
Median BNP level (IQR), pg/ml	208.7 (104.0–513.1)	437.5 (124.9–751.2)	0.147
Median left ventricular ejection fraction (IQR), %	50 (41–57)	44 (34–53)	0.172
Median estimated GFR (IQR), ml/min/1.73 m^2^	62.5 (48.8–79.0)	51.5 (37.5–71.8)	0.074
Peak V̇O2 at 2 months	11.7 ± 1.9	12.0 ± 1.4	0.682
Peak V̇O2 at 5 months	15.1 ± 3.5	10.8 ± 1.8	< 0.001
**NYHA class -N. (%)**			0.457
I	55 (64)	12 (50)	
II	24 (28)	9 (38)	
III	7 (8)	3 (13)	
**Coexisting conditions, N (%)**			
Hypertension	58 (67)	13 (54)	0.239
Diabetes	30 (35)	8 (33)	1.000
Atrial fibrillation	15 (17)	2 (8)	0.354
Prior thromboembolic events	2 (2)	1 (4)	0.526
CHADS2 score (IQR)	2 (2–3)	2 (2–3)	0.391
CHA2DS2-VASc (IQR)	4 (3–5)	4 (3–5)	0.673
Heart failure with preserved ejection fraction, N(%)	45 (52)	8 (33)	0.112
**Cause of heart failure, N (%)**			1.000
Ischemic	63 (73)	17 (71)	
Non-ischemic	8 (9)	6 (25)	
Idiopathic	15 (17)	1 (4)	
Valvular			
**Medications, N (%)**			
ACE inhibitor or ARB	77 (90)	23 (96)	0.688
Beta-blocker	71 (83)	16 (67)	0.153
Amiodarone	5 (6)	1 (4)	1.000
Preexisting pacemaker or CRT, N (%)	3(4)	0	1.000

IQR; interquartile range, GFR; glomerular filtration rate, NYHA; New York Heart Association, ACE; angiotensin-converting enzyme, ARB; angiotensin II receptor blocker, CRT; cardiac resynchronization therapy, BNP; brain natriuretic peptide.

### Long-term outcomes

**Figure 1:**
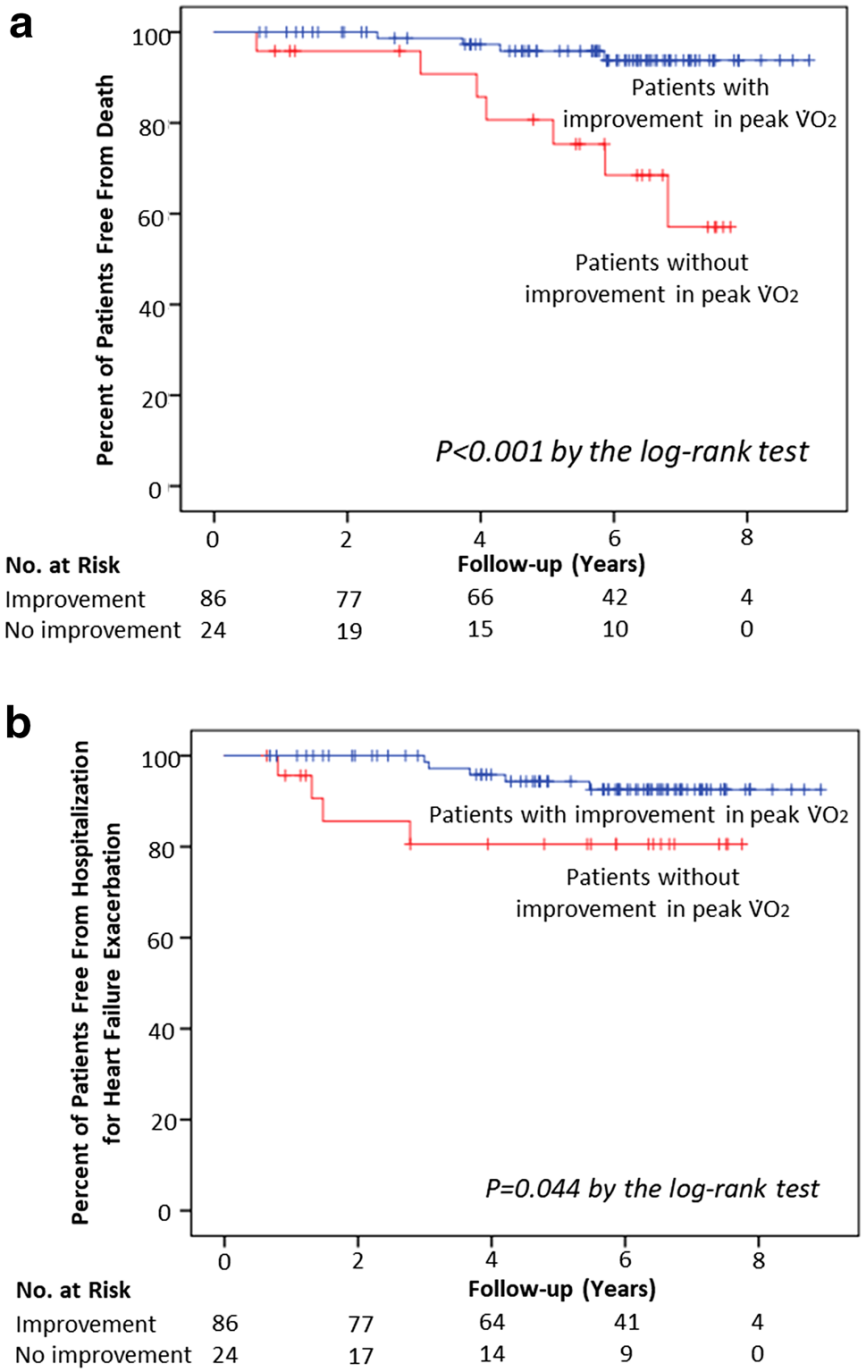
Mortality (**a**) and hospitalization due to heart failure exacerbation (**b**) over time. The Kaplan–Meier estimates of mortality and hospitalization for HF exacerbation over a median follow-up of 6 years are shown for high-risk patients with improvement in peak V̇O_2_, high-risk patients without improvement in peak V̇O_2_, and patients with a preserved V̇O_2_. Tick marks indicate censored data. HF, heart failure; V̇O_2_, peak exercise oxygen consumption.

**Figure 2:**
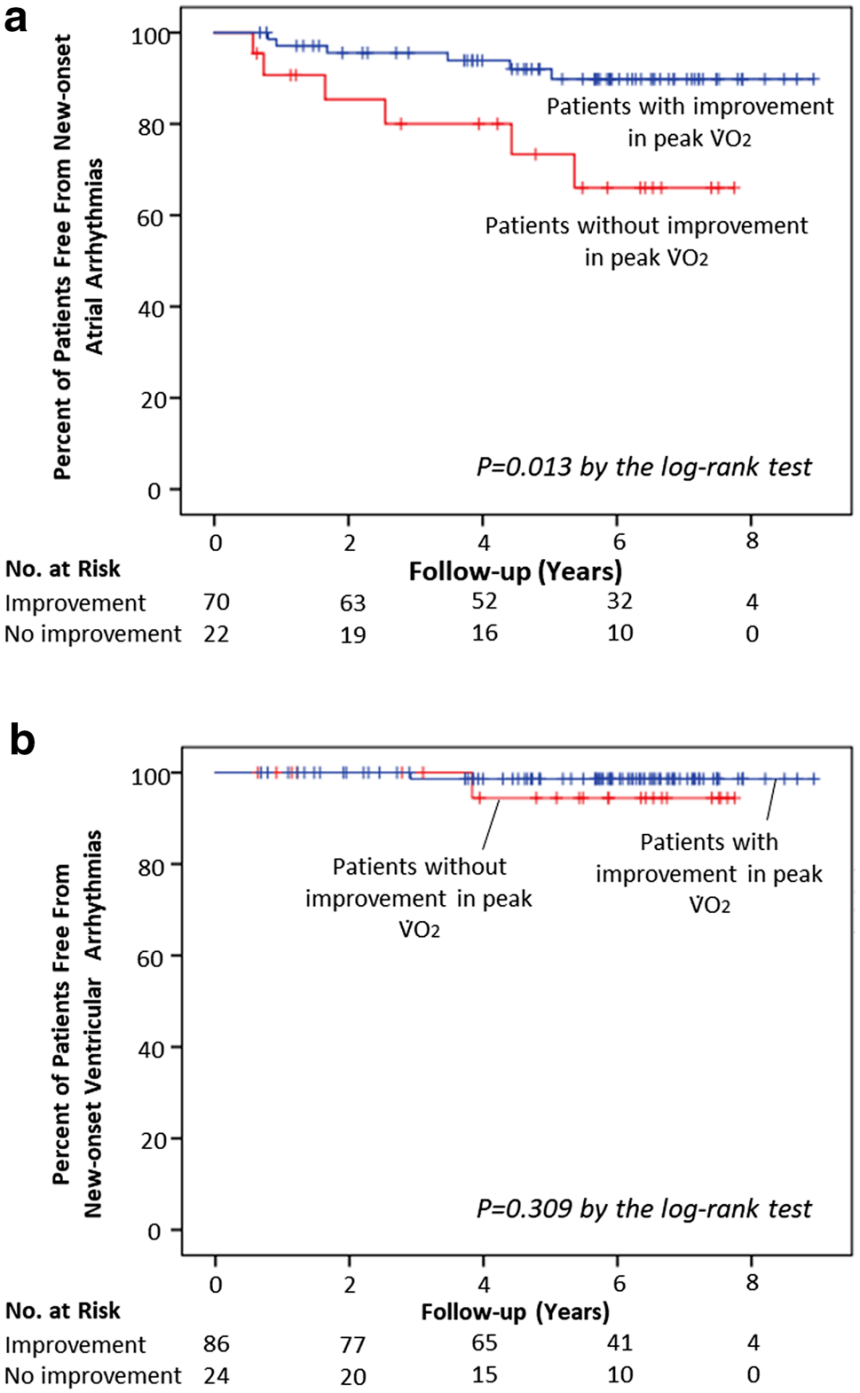
New-onset atrial (**a**) and ventricular (**b**) arrhythmias over time. The Kaplan–Meier estimates of the incidence of new-onset atrial and ventricular arrhythmias over a median follow-up of 6 years are shown for high-risk patients with improvement in peak V̇O_2_, high-risk patients without improvement in peak V̇O_2_, and patients with a preserved V̇O_2_. Tick marks indicate censored data. HF, heart failure; V̇O_2_, peak exercise oxygen consumption.

During a median follow-up of 6 years, higher-risk patients with improvement in peak V̇O_2_, compared to those without peak V̇O_2_ improvement, had a lower rate of mortality (4% vs. 29%; log-rank, *P* < 0.001, Fig. 1a) and hospitalization due to HF exacerbation (6% vs. 17%; log-rank, *P* = 0.044, Fig. 1b). Unadjusted hazard ratios of mortality and hospitalization for higher-risk patients with an improved peak V̇O_2_ with CR were 0.132 (95% confidence interval [CI] 0.034–0.511; *P* = 0.003) and 0.156 (95% CI 0.018–0.1373; *P* = 0.094), respectively. Among 196 patients without prior AF, 23 patients (12%) had atrial arrhythmias: AF in 18 patients and atrial tachycardia in five. Higher-risk patients with improvement in peak V̇O_2_ with CR had a lower incidence of new-onset atrial arrhythmias than did those without a peak V̇O_2_ improvement (9% vs. 27%; log-rank, *P* = 0.013, Fig. 2a). Unadjusted hazard ratio of new-onset atrial arrhythmias for patients with an improved peak V̇O with CR was 0.263 (95% CI 0.085–0.830; *P* = 0.021). New-onset ventricular arrhythmias occurred in four patients: ventricular tachycardia in three patients and ventricular fibrillation in one. There was no difference in the incidence of ventricular arrhythmias between higher-risk patients with and without improvement in peak V̇O_2_ with CR (1% vs. 4%; log-rank, *P* = 0.309, Fig. 2b). Unadjusted hazard ratio of new-onset ventricular arrhythmias for higher-risk patients with an improved peak V̇O_2_ with CR was 0.262 (95% CI 0.016–4.195; *P* = 0.344). Patients with peak V̇O_2_ percentage of predicted values of < 45% had a higher risk of mortality (log-rank, *P = *0.010) and higher incidence of new-onset atrial arrhythmias than did those with peak V̇O_2_ percentage of predicted values of ≥ 45% (log-rank, *P* = 0.031), though no differences were found in hospitalization due to HF exacerbation (log-rank, *P* = 0.081) and new-onset ventricular arrhythmias (log-rank, *P* = 0.229).

Table 3 summarizes the causes of death among the 11 patients with a low peak V̇O_2_. Eight of these 11 patients died from cardiovascular events (73%), with progressive HF being the predominant cause (6 of 11 patients, 55%). In these cases, there was no incidence of thromboembolic events. Of the eight patients who died from cardiovascular events, three had new-onset atrial arrhythmias (38%). None of the higher-risk patients underwent catheter ablation for new-onset atrial arrhythmias.

**Table 3:** Causes of death in the higher-risk patients without improvement in peak V̇O2.

	No. (%)
Total	11
Cardiovascular death	8 (73)
**Cardiac**	
Sudden cardiac death	2(18)
Progressive heart failure	6 (55)
**Vascular**	
Thromboembolic events	0
Hemorrhage	0
**Non-cardiovascular death**	3 (27)
Cancer	1 (9)
Infection	2 (18)

## Discussion

Our major findings were as follows: (1) the majority of patients with a low peak V̇O_2_ (higher-risk patients), defined as < 14 mL/min/kg, had improvement in peak V̇O_2_ at 3 months after CR; (2) higher-risk patients with an improved peak V̇O_2_ had a lower rate of mortality and hospitalization due to HF exacerbation, as well as a lower incidence of new-onset atrial arrhythmias than did those without improvement in peak V̇O_2_, but with no difference in the incidence of ventricular arrhythmias between groups; (3) patients with peak V̇O_2_ percentage of predicted values of < 45% had a higher risk of mortality (log-rank, *P* = 0.010) and higher incidence of new-onset atrial arrhythmias than did those with peak V̇O_2_ percentage of predicted values of ≥ 45% (log-rank, *P* = 0.031); and (4) the majority of deaths in higher-risk patients were cardiovascular events, with progressive HF being predominant.

Impaired exercise capacity is a major symptom in patients with HF, which is represented by low peak V̇O_2_ during maximal exercise testing. A previous study by Mancini et al. demonstrated that a low peak V̇O_2_ of < 14 mL/kg/min was associated with poor prognosis in patients with severe HF^1^. A comprehensive CR program includes patient evaluation, exercise training, physical activity counseling, cardiovascular risk factor management, psychosocial support, and patient education^3,5^. Previous studies demonstrated that CR improved peak V̇O_2_ and decreased all-cause mortality in patients with HF^5,9,11–13^. The HF-ACTION trial demonstrated the positive impact of CR on the long-term cardiovascular mortality or HF hospitalization during a median follow-up of 30 months^13^, which was in accordance with our findings. However, long-term outcomes of higher-risk patients without improvement in peak V̇O_2_ despite a comprehensive CR program have not been fully investigated, particularly regarding the long-term incidence of atrial and ventricular arrhythmias in those patients. In this study, higher-risk patients without improvement in peak V̇O_2_ after CR had a higher rate of mortality, HF hospitalization, and new-onset atrial arrhythmias than those with improvement. Of note, the majority of deaths were progressive HF, suggesting that most patients with severe HF, who are more prone to develop atrial arrhythmias, easily evolve towards advanced HF.

HF predisposes to the occurrence of atrial arrhythmias through various mechanisms, including an increase in left ventricular filling pressure or left atrial dilatation and fibrosis, each of which can lead to atrial structural and electrical remodeling^14^. Conversely, atrial arrhythmias, with an increased heart rate, can predispose to the development or worsening of HF due to impaired contractility and reduced cardiac output. Therefore, the occurrence of atrial arrhythmias in patients with HF is associated with increased adverse events, including HF progression and mortality^15,16^. In our study, the majority of deaths of higher-risk patients without improvement in peak V̇O_2_, who were at high risk for new-onset atrial arrhythmias, were cardiovascular events, mostly progressive HF, which was in accordance with previous studies. Although the treatment of atrial arrhythmias in patients with HF is of pivotal importance, previous randomized trials have failed to demonstrate that maintenance of sinus rhythm with antiarrhythmic drug therapy improves mortality in patients with coexisting HF and AF^17^, suggesting that side effects of antiarrhythmic drugs offset the positive effects. Recent studies have shown that catheter ablation is associated with a positive outcome in comparison with antiarrhythmic drugs^18–20^. The CASTLE-AF (Catheter Ablation versus Standard Conventional Therapy in Patients with Left Ventricular Dysfunction and Atrial Fibrillation) trial showed that catheter ablation was associated with lower rates of death from any cause and lower rates of hospital admission for HF in patients with HF. In a sub-analysis of the CAMERA MRA study, a regression of ventricular fibrosis in the context of reverse remodeling was observed on cardiac magnetic resonance imaging after restoration of sinus rhythm following catheter ablation of AF in patients with HF^21^, suggesting that timely treatment with catheter ablation may minimize irreversible ventricular remodeling. Although none of the deceased higher-risk patients with new-onset atrial arrhythmias underwent catheter ablation in this study, catheter ablation at the early stage of new-onset atrial arrhythmias may have improved the long-term outcome^22^.

## Limitations

First, this was a retrospective single-center study with a limited number of patients since we aimed to include those who were able to be followed for a long-term period, which was possibly associated with the low incidence of new-onset ventricular arrhythmias. Furthermore, due to the limited number of patients, multivariate analysis was not performed in this study. Further multicenter investigation in a large number of patients is warranted. Second, as in many other countries, the number and duration of supervised CR sessions were limited due to a reimbursement policy in Japan. The prolonged CR may have further improved peak V̇O_2_, as observed in a previous study^23^, particularly in higher-risk patients. Third, considering that most patients enrolled in this study were on NYHA class I, with optimal therapies at the 2-month time point of CR, our data can be applied only to patients without severe HF who are refractory to medical and interventional therapies. Fourth, due to the lack of a predefined protocol in this retrospective study, systematic follow-up echocardiographic evaluation was not performed in this study to assess the relationship between the peak V̇O_2_ improvement and changes in echocardiographic parameters.

## Conclusion

Improvement in peak V̇O_2_ after CR reduced the risk of new-onset atrial arrhythmias, mortality, and hospitalization due to HF exacerbation in higher-risk patients with HF. Further studies are warranted to determine whether early detection and management at the early stage of new-onset atrial arrhythmias could improve clinical outcomes in higher-risk patients without improvement in exercise capacity after CR.

## Data Availability

The datasets generated during and/or analyzed during the current study are available from the corresponding author on reasonable request.
